# Evaluation of Muscle Oxygen Dynamics in Children’s Gait and Its Relationship with the Physiological Cost Index

**DOI:** 10.3390/healthcare11020221

**Published:** 2023-01-11

**Authors:** Yuya Shirai, Tadashi Ito, Yuji Ito, Naomichi Matsunaga, Koji Noritake, Nobuhiko Ochi, Hideshi Sugiura

**Affiliations:** 1 Department of Integrated Health Sciences, Nagoya University Graduate School of Medicine, Nagoya 461-8673, Japan; 2 Department of Rehabilitation, Nagoya University Hospital, Nagoya 466-8560, Japan; 3 Three-Dimensional Motion Analysis-Laboratory, Aichi Prefectural Mikawa Aoitori Medical and Rehabilitation Center for Developmental Disabilities, Okazaki 444-0002, Japan; 4 Department of Pediatrics, Nagoya University Graduate School of Medicine, Nagoya 466-8560, Japan; 5 Department of Pediatrics, Aichi Prefectural Mikawa Aoitori Medical and Rehabilitation Center for Developmental Disabilities, Okazaki 444-0002, Japan; 6 Department of Orthopedic Surgery, Aichi Prefectural Mikawa Aoitori Medical and Rehabilitation Center for Developmental Disabilities, Okazaki 444-0002, Japan

**Keywords:** muscle oxygen saturation, near-infrared spectroscopy, physiological cost index, children, gait

## Abstract

The response of muscle oxygen saturation, which is an index for the energy metabolism of muscles during walking in children, and its relationship to the physiological cost index, which indicates walking efficiency, are unknown. This study aimed to evaluate muscle oxygen saturation in lower extremity muscles during walking in children, its changes with age, and the relationship between the physiological cost index. The oxygen saturation was measured by the amount of change during a two-minute walk, and the physiological cost index was calculated from the change in heart rate before and after exercise and walking speed. Results were compared for each muscle, and the correlation between the two was examined. Changes in muscle oxygen saturation were greater in the lower leg muscles, significantly greater in the tibialis anterior at six to seven years, and in the gastrocnemius medial head at eight to ten years. The physiological cost index was significantly correlated with changes in muscle oxygen saturation in the tibialis anterior (r = 0.44, *p* < 0.001). The lower leg muscles were metabolically active in children’s gait, and their response varied with age. Moreover, the muscle oxygenation dynamics of the tibialis anterior may influence walking efficiency.

## 1. Introduction

Energy metabolism is an essential bodily function, but its response is determined by the developmental stage of the individual [1]. In prepubertal children, body and muscle mass grow with age [2,3] and change oxygen consumption and requirements [4]. These findings suggest that age-related changes in energy metabolism response occur with development. However, there is a lack of information on the energy metabolism response of muscle and how it changes with age.

Studies have focused on muscle oxygen dynamics as an energy metabolism response in skeletal muscle [5,6,7]. Muscle oxygen saturation (SmO_2_), obtained by near-infrared spectroscopy (NIRS), has been investigated as a means of evaluating muscle oxygen dynamics [8,9]. The change in SmO_2_ during exercise is a useful indicator for assessing muscle energy metabolism in terms of muscle oxygen consumption during muscle activity. It was reported that the muscle activity pattern of children’s gait changes at around seven years of age [10,11], suggesting that the muscles of children of different ages will demonstrate different SmO_2_ values during gait. Although a mature gait pattern in children may be established early by age six to eight years on average, fine control of gait performance, which is a joint activity of the main agonist and antagonist muscles and motor switching of individual muscles, continues to develop over the years [11,12,13].

Children who participate in physical training have a high muscle energy metabolism and experience exercise-induced fatigue more slowly [14]. In contrast, impaired muscle energy metabolism results in poor exercise performance [15]. Muscle fatigue and poor performance are primarily associated with the depletion of energy substrates such as muscle glycogen and the livestock nature of metabolic waste products [16]. Compared with adults, children have a lower anaerobic capacity and are more susceptible to energy depletion [17,18,19]. Moreover, oxygen-based metabolism in muscle is an important compensatory mechanism in energy production during exercise in children, although it is more energy inefficient when oxygen is depleted. Therefore, it may be valuable to identify signs of deteriorating muscle energy metabolism and walking efficiency to understand children’s exercise performance. Thus, quantifying how muscles respond to physical exercise is of great interest for the study of muscle energy metabolism and walking efficiency in children for improved performance. Little data exist on the effects of growth on walking efficiency and muscle energy metabolism. Additionally, few studies have evaluated the SmO_2_ of each muscle in children’s gait and its difference with age.

One way of ascertaining walking efficiency is the physiological cost index (PCI) [20,21,22]. Clarifying the relationship between walking efficiency in terms of energy consumption and the energy metabolism of each muscle will facilitate the development of more effective rehabilitation interventions.

This study aimed to evaluate the SmO_2_ of the lower limb muscles during walking in children and the differences in response according to age to determine the relationship between SmO_2_ and PCI, which indicates walking efficiency. Since boys and girls show differences in physical development during puberty, it is necessary to consider the effects of age. Therefore, prepubescent children in the lower and middle grades of elementary school were the focus of this study.

This study investigated two hypotheses for the lower limb muscles of children during walking: (1) The response of SmO_2_ varies with muscle and age, and (2) the energy metabolism of each muscle correlates with walking efficiency.

## 2. Materials and Methods

### 2.1. Research Design and Subjects

This prospective observational study was conducted from October 2019 to December 2020 among elementary school students living in Okazaki City, Aichi Prefecture, Japan, during the Okazaki medical health check-up for children. The recruitment method was to distribute flyers at two elementary schools introduced by the Okazaki City Board of Education, and 97 children aged six to ten who wanted to participate were targeted. The exclusion criteria were a history of neuromuscular, cardiovascular/respiratory, and metabolic diseases, and any previous orthopedic diagnosis. After excluding two children with a history of lower limb fracture, three with a history of cardiovascular diseases, four with a history of respiratory diseases, and 29 for whom SmO_2_ or PCI could not be calculated due to missing data, a total of 59 children were included in the analysis. The final study participants were 28 boys and 31 girls, ranging in age from six to ten years (mean age 7.3 ± 1.2 years).

In prepubertal children, oxygen consumption increases with age [23], and body mass and muscle mass grow with age [2,3]. Furthermore, the observed relationship between maximal oxygen uptake and lean body mass [24] may change with development. In addition, changes in muscle activity patterns, including co-contraction and reciprocal contraction [10,11] and gait patterns are established [12,13], suggesting that the energy metabolism response of muscle at this age may also differ. Therefore, in this study, to examine the effects of age and sex on SmO_2_, the children were classified into four groups based on their age and sex. The four groups comprised those under seven years and those over eight years old when categorized by age, and younger-age boys (YA boys), older-age boys (OA boys), younger-age girls (YA girls), and older-age girls (OA girls) when categorized by sex. In this study, younger and older age refer to six- to seven-year-olds and eight- to ten-year-olds, respectively.

### 2.2. Measurement Details and Methods

#### 2.2.1. Physical Information

The participants’ height, weight, body fat percentage, and skeletal muscle mass were measured using the multi-frequency, three-electrode bioelectrical impedance analysis method with a TANITA MC-780 body composition analyzer (TANITA Corporation, Tokyo, Japan). The target age group was six years and older, which is the same as the target age used in the previous study [25]. Skeletal muscle mass was defined as the sum of the muscle masses of all four limbs. Body mass index and skeletal muscle mass index were calculated from the obtained body weight and skeletal muscle mass data by dividing each value by the square of its height. The results are presented in Table 1. Bioelectrical impedance analysis was performed 2 h after meals [26].

#### 2.2.2. Measurement of Muscle Oxygen Dynamics

The NIRS instrument used was a Moxy Muscle Oxygen Monitor (Moxy Monitor, Minnesota, USA). This device [27,28,29,30,31] uses near-infrared light emitted from the light transmitter of a probe attached to the skin on the surface of the target muscle. Near-infrared light is detected by the light receiver after being absorbed and scattered as it passes through the skin, subcutaneous fat, and muscle. The amount of light absorbed is calculated as the logarithm of the intensity entering the sample divided by the intensity exiting the sample. Using Beer–Lambert’s law, the concentration of a mixture of chromophores can be determined. From the wavelength of the detected light, it is possible to detect oxidized hemoglobin bound to serum hemoglobin and oxygen in microvessels (small arteries, capillaries, and veins), as well as oxidized myoglobin bound to myoglobin and oxygen in the muscle cytoplasm, allowing quantification of the respective changes. Using these detection items, SmO_2_ is calculated by the following formula:

((oxidized hemoglobin + oxidized myoglobin)/(total amount of hemoglobin + myoglobin)) × 100%

SmO_2_ is used as an index that reflects the dynamic balance between oxygen supplied in the circulation by microvessels and oxygen consumption by the muscles [28,29,30,31,32]. SmO_2_ measurements have been made during various walking tasks in previous studies, and this method is also used to evaluate the dynamic contraction of muscles during walking [33,34,35].

The muscles included in this study were the rectus femoris (RF) and semimembranosus-semitendinosus (SS) in the thigh and the tibialis anterior (TA) and gastrocnemius medial head (GS) in the lower leg. The NIRS probes were then applied to the belly of the right lower extremity muscle of each participant. The NIRS probes were placed in the positions recommended by Surface ElectroMyoGraphy for the Non-Invasive Assessment of Muscles (SENIAM) [36] as follows: RF, 50% on the line from the anterior spina iliaca superior to the superior part of the patella; SS, 50% on the line between the ischial tuberosity and the medial epicondyle of the tibia; TA, proximal 33% of the line between the tip of the fibula and medial malleolus; GS, proximal 33% of the line between the medial epicondyle of the femur and calcaneus.

**Figure 1:**
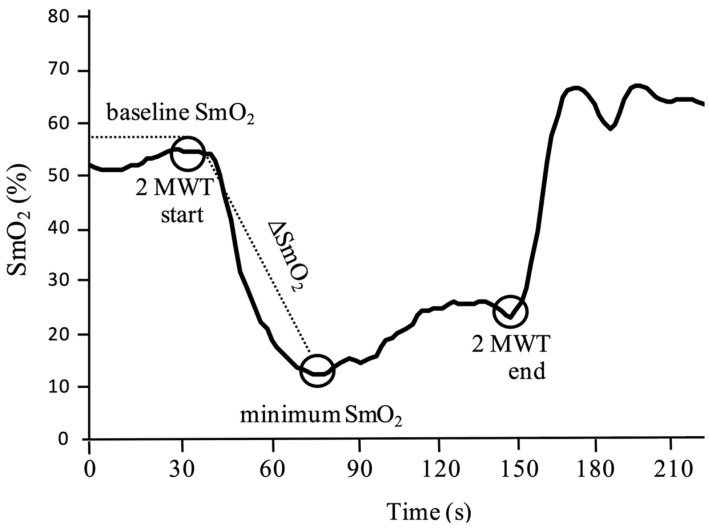
Calculation method for SmO_2_ parameters obtained before and during the 2 MWT. The average SmO_2_ value during the 30 s of rest before the start of exercise was defined as the baseline, then the minimum value of SmO_2_ during exercise was calculated. The difference between these values was defined as ΔSmO_2_.

We conducted a two-minute walk test (2 MWT) with optimal walking and obtained SmO_2_ data using NIRS, referring to previous studies [8,9,32]. From the data, we calculated the average value of SmO_2_ during the 30 s rest period before the start of exercise (baseline [%]), the minimum value of SmO_2_ during walking (minimum [%]), and the difference between the baseline and minimum values (ΔSmO_2_ [%]) as an indicator of the load on the muscle. A schematic of the calculation method is shown in Figure 1.

#### 2.2.3. Physiological Cost Index

PCI is calculated based on changes in heart rate and walking speed. PCI occurs during walking in healthy children [37]. We calculated PCI from the 2 MWT of optimal walking, heart rate during walking, resting heart rate, and walking speed measurements. The 2 MWT was preceded by a three-minute resting period before the test. In the 2 MWT, participants walked both ways on a 10 m straight walking path following a standardized protocol. The participants were allowed to stop and rest, and the time-lapse was continued [38]. However, none of the participants rested. The 2 MWT has been used to meaure gait efficiency in previous studies [26], and its validity has been demonstrated [39]. PCI was measured using a POLAR M 600 (Polar Electro, Tokyo, Japan), a sports-optimized smartwatch designed to measure heart rate. The smartwatch was attached to the participant’s right wrist. PCI [20,21,22] was calculated with the following formula: PCI = (heart rate during walking—resting heart rate)/walking speed.

### 2.3. Statistical Analysis

The optimal sample size for Spearman’s rank correlation analysis was determined through a power analysis using G*Power software (Heinrich Heine University Düsseldorf, Düsseldorf, Germany) [40,41]. For this analysis, alpha was set to 0.05, the statistical power to 0.80, and the effect size on two-tailed tests to 0.5. This result indicated a required sample size of 26 participants.

Statistical analysis was performed with R version 4.0.3 (R Foundation for Statistical Computing, Vienna, Austria). Descriptive statistics are presented as mean ± standard deviation, median, and interquartile range. The Shapiro–Wilk test was performed to check for the normality of the data obtained. The Kruskal–Wallis test was performed to compare the muscle oxygen dynamic assessment (ΔSmO_2_) of each muscle measured by NIRS between the four groups. The Steel–Dwass test was performed to compare ΔSmO_2_ per muscle in each group as a multiple comparison test. In addition, Spearman’s rank correlation coefficient was used to determine the association between PCI and ΔSmO_2_ of each muscle in all children. Statistical significance was set at *p* < 0.05.

## 3. Results

### 3.1. Muscle Oxygen Dynamics during Walking in Each Muscle

**Figure 2:**
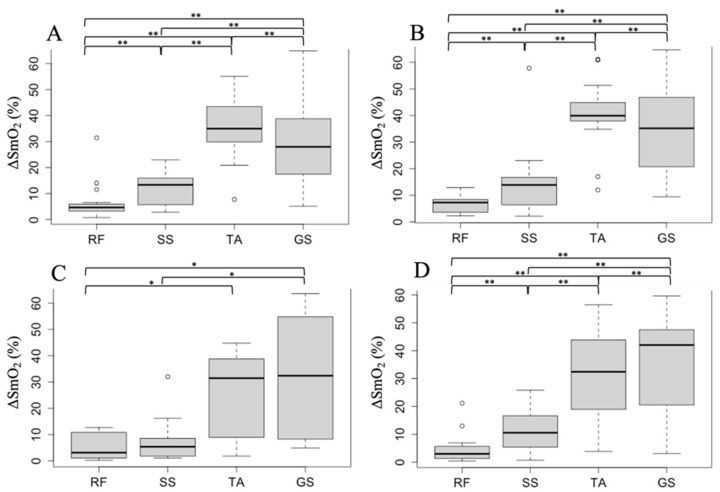
Comparison of the participants’ ΔSmO_2_ in each group. Data were analyzed using the Steel–Dwass test. (**A**) In younger-age (YA) boys, a significant difference was found in the ΔSmO_2_ of all muscles (*p* < 0.001). The ΔSmO_2_ of the TA was highest among all the muscles. (**B**) In YA girls, a significant difference was found in the ΔSmO_2_ of all muscles (*p* < 0.001). The ΔSmO_2_ of the TA was highest among all the muscles. (**C**) In older-age (OA) boys, ΔSmO_2_ was significantly larger in the TA and GS than in the RF, and ΔSmO_2_ was significantly larger in the GS than in the SS (*p* < 0.05). No significant difference was found in ΔSmO_2_ between the TA and GS. (**D**) In girls with OA, a significant difference was found in the ΔSmO_2_ of all muscles (*p* < 0.001). The ΔSmO_2_ of GS was highest among all the muscles. An asterisk (*) indicates a statistically significant (*p* < 0.05), and double asterisks (**) indicate a statistically significant (*p* < 0.001).

The baseline, minimum, and ΔSmO_2_ during walking in each muscle are listed in Table 2. In the four groups of YA boys, OA boys, YA girls, and OA girls, ΔSmO_2_ was significantly different according to the Kruskal–Wallis test (*p* < 0.01). The results of the Steel–Dwass test showed that ΔSmO_2_ was significantly higher in the lower leg muscles than in the thigh muscles in all four groups (see Figure 2). Regarding the muscles of the lower leg, ΔSmO_2_ of TA was significantly higher than that of GS (34.9% vs. 27.9%, *p* < 0.001) in the YA boys, while no significant difference was observed in the OA boys. The ΔSmO_2_ of TA was significantly higher than that of GS (39.9% vs. 35.1%, *p* < 0.001) in the YA girls, while the ΔSmO_2_ of GS was significantly higher than that of TA (42.0% vs. 32.4%, *p* < 0.001) in the OA girls (see Figure 2).

### 3.2. Association between PCI and ΔSmO_2_ during Walking

**Figure 3:**
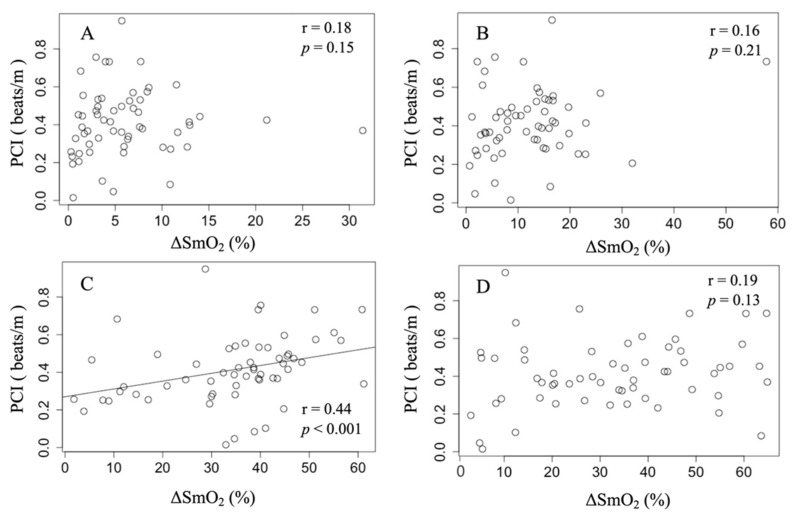
Correlation between PCI and ΔSmO_2_ in each muscle. The data were analyzed using Spearman’s rank correlation coefficient. (**A**): No significant differences were found among the PCI and ΔSmO_2_ values in the RF of all children (r = 0.18, *p* = 0.15). (**B**): No significant difference was found among the PCI and ΔSmO_2_ values in SS in all children (r = 0.16, *p* = 0.21). (**C**): There was a significant difference between PCI and ΔSmO_2_ in the TA of all children with a moderately positive correlation (r = 0.44, *p* < 0.001). (**D**): No significant difference was found between PCI and ΔSmO_2_ in the GS of all children (r = 0.19, *p* = 0.13).

Table 3 shows the results of the PCI. A significant moderate correlation was observed between PCI and ΔSmO_2_ of the TA during walking in all children (r = 0.44, *p* < 0.001). However, no correlation was observed between PCI and ΔSmO_2_ for the RF, SS, or GS (see Figure 3).

## 4. Discussion

In this study, NIRS was used to evaluate muscle oxygen dynamics in the lower limbs during walking and to evaluate our hypothesis. Our results showed that the lower leg muscles were subjected to a greater load of energy metabolism than the thigh muscles during walking. Furthermore, the muscle oxygen dynamics in the lower leg muscles differed between the age groups. To our knowledge, this is the first study to show a relationship between walking efficiency and muscle oxygen dynamics in the TA.

The results revealed significant differences in the muscle-by-muscle comparison of ΔSmO_2_ during walking. In particular, ΔSmO_2_ was higher in the lower leg muscles than in the thigh muscles. These results support reports that RF and SS slightly contribute to walking in adults [42,43], that muscle activity around the hip and knee joints is low, and that activity around the ankle joints is high during walking in children [43]. The results of this study are similar to those of previous studies [42,43,44], as ΔSmO_2_ was larger in the lower leg muscles than in the thigh muscles because of the higher muscle activity level and greater intramuscular oxygen consumption of TA and GS in the ankle joint muscles. In addition, previous studies using PET scans showed that a large metabolism response of each muscle in gait occurs in the triceps surae muscles, such as the soleus and GS, followed by high TA and low metabolic activity in the RF and SS [45,46]. Therefore, it is likely that the lower leg muscles are similarly greatly involved in walking in children aged six to ten years. Furthermore, ΔSmO_2_ of TA was larger in YA and ΔSmO_2_ of GS was larger in OA, indicating that the activation reaction of muscles in the lower legs during walking may be different in YA and OA. This may be due to changes in the muscle activity patterns in gait that occur with development. On average, gait patterns [12,13] and muscle activity patterns evaluated by electromyography [10,11] mature at six to eight years old. However, given that SmO_2_ indicates muscle activation in terms of metabolism response [47], it is suggested that the YA group in this study may have responded differently to the OA group because their muscle activity patterns were not fully mature. This response may be influenced by the joint contraction of the lower leg muscles when the muscle activity pattern is not fully matured [11]. In short, it is suggested that TA muscle activity and its metabolism response may not be fully mature in six- to seven-year-old children. Furthermore, the results of the OA group also fit with the concept that the parts of the triceps surae muscle that are the most metabolically active in gait are also identified in other studies [45,46]. The GS was likely to be the most metabolically active of the target muscles because it is more involved in kicking and forward propulsion [48,49].

A significant, moderately positive correlation was observed between PCI and ΔSmO_2_ of the TA. A higher value of PCI is interpreted as poor walking efficiency, and PCI is affected by the muscle activity of the lower extremities [50]. Thus, based on the present results, we can say that the larger the value of ΔSmO_2_ in the TA (or the larger the amount of load applied to the TA), the lower the walking efficiency. This may be because the greater the muscle activity of the TA and associated metabolic reactions occurring during walking in children, the more energy is required and the lower the energy efficiency during waking. We believe that to bring PCI closer to normal, the amount of load on the TA during walking should not be increased too much, and coordinated motor training of the lower extremities should be performed so that the GS is most activated. This is so that the lower leg muscles can be used and coordinated during walking. Therefore, evaluating the energy metabolism and efficiency of each muscle may be an effective rehabilitation intervention for children with reduced walking efficiency. However, further studies should be conducted on the changes in muscle coordination and metabolism response of the lower leg during walking and in relation to walking efficiency to determine whether to reduce the load on the TA during walking.

## 5. Limitations

Some of the limitations of this study are as follows. First, the thickness of the subcutaneous fat just below the probe may interfere with the measurement of SmO_2_. Since we did not measure the thickness of the subcutaneous fat in this study, we could not account for this effect, which may have been underestimated. Second, we were not able to evaluate muscle activity using electromyography. Therefore, it is not possible to compare the difference between the energy metabolism response in muscle activity and muscle activity using electromyography. Finally, the sample size of the study was small, and the results from a limited group of subjects are difficult to generalize. Therefore, we believe a larger sample size will allow more generalizable results. As the participants in this study were exclusively Japanese, the results of this study should only be applied to the Japanese population, and it is difficult to justify their application to various populations around the world.

## 6. Conclusions

The findings of this study suggest that energy metabolism in children’s gait involves the muscles of the lower leg more than those of the thigh. Among them, it was shown that the response of the lower leg muscles might vary by age group. Moreover, the muscle oxygen dynamics of the TA may affect the walking efficiency determined by PCI.

## Figures and Tables

**Table 1:** Demographic characteristics of participants.

	YA Boys (*n* = 18)	OA Boys (*n* = 10)	YA Girls (*n* = 18)	OA Girls (*n* = 13)
Age (years)	6.3 ± 0.4	9.0 ± 0.6	6.5 ± 0.5	8.5 ± 0.6
Height (cm)	119.1 ± 5.1	134.0 ± 5.9	118.1 ± 4.5	129.3 ± 6.6
body weight (kg)	21.3 ± 4.2	27.8 ± 5.2	22.2 ± 3.4	25.9 ± 3.7
BMI (kg/m^2^)	14.9 ± 2.3	15.3 ± 1.7	15.8 ± 1.6	15.3 ± 0.8
Body fat (%)	10.2 ± 7.3	10.1 ± 4.7	15.0 ± 4.6	13.3 ± 3.0
SMI (kg/m^2^)	5.3 ± 0.4	5.6 ± 0.4	5.3 ± 0.3	5.4 ± 0.2
2 MWD (m)	119.7 ± 14.5	129.1 ± 22.7	113.6 ± 10.5	122.9 ± 13.9

Data are presented as mean ± standard deviation. Abbreviations: YA: younger age; OA: older age; BMI: body mass index; SMI: skeletal muscle mass index; 2 MWD: two-minute walk distance.

**Table 2:** The SmO_2_ values for each muscle in the four groups.

YA Boys					
	RF	SS	TA	GS	*p*-Value
Baseline (%)	65.9 [60.7–70.7]	61.2 [55.3–72.1]	48.8 [46.4–52.2]	54.9 [51.8–60.2]	-
Minimum (%)	58.9 [57.2–63.0]	50.9 [47.1–55.7]	12.7 [7.8–22.0]	23.8 [15.9–41.2]	-
ΔSmO_2_ (%)	4.6 [3.3–5.9]	13.3 [6.3–15.7]	34.9 [29.9–43.2]	27.9 [17.5–18.0]	<0.001
**OA Boys**					
	**RF**	**SS**	**TA**	**GS**	***p*-Value**
Baseline (%)	64.3 [59.1–75.7]	56.0 [52.0–60.1]	51.2 [48.9–53.6]	58.8 [49.7–62.3]	-
Minimum (%)	63.5 [53.0–71.6]	48.8 [43.9–53.3]	23.5 [11.2–34.7]	23.1 [7.3–41.4]	-
ΔSmO_2_ (%)	3.1 [1.1–10.0]	5.4 [1.9–8.4]	31.4 [10.3–37.7]	32.3 [12.9–50.9]	0.001
**YA Girls**					
	**RF**	**SS**	**TA**	**GS**	***p*-Value**
Baseline (%)	73.9 [61.4–81.6]	67.6 [62.6–75.7]	53.4 [47.1–67.6]	52.5 [50.7–60.3]	-
Minimum (%)	65.8 [57.8–74.2]	52.6 [47.8–60.2]	12.1 [6.0–27.6]	17.1 [13.5–31.9]	-
ΔSmO_2_ (%)	7.2 [3.7–8.2]	13.8 [6.7–16.5]	39.9 [38.1–44.0]	35.1 [21.4–46.5]	<0.001
**OA Girls**					
	**RF**	**SS**	**TA**	**GS**	***p*-Value**
Baseline (%)	66.8 [65.6–72.7]	65.3 [57.7–71.4]	52.3 [47.8–58.1]	55.7 [49.1–59.3]	-
Minimum (%)	62.2 [59.8–67.8]	52.4 [42.9–59.5]	16.0 [7.4–35.0]	12.2 [6.9–32.7]	-
ΔSmO_2_ (%)	2.9 [1.3–5.6]	10.5 [5.4–16.6]	32.4 [18.9–43.8]	42.0 [20.5–47.5]	<0.001

The results are presented as median [Interquartile range]. The data of ΔSmO_2_ of each muscle in each group were analyzed using Kruskal–Wallis test. Abbreviations: YA: younger age; OA: older age; RF: rectus femoris; SS: semimembranosus-semitendinosus; TA: tibialis anterior; GS: gastrocnemius medial head; ΔSmO_2_: Δ muscle oxygen saturation.

**Table 3:** The physiological cost index values for the four groups.

	YA Boys (*n* = 18)	OA Boys (*n* = 10)	YA Girls (*n* = 18)	OA Girls (*n* = 13)
PCI (beats/m)	0.45 ± 0.16	0.23 ± 0.14	0.46 ± 0.17	0.43 ± 0.13

Data are presented as mean ± standard deviation. Abbreviations: YA: younger age; OA: older age; PCI: physiological cost index.

## Data Availability

All of the relevant data are presented within the manuscript. All data are available from the authors on request.
